# Anti-cancer Drugs Associated Atrial Fibrillation—An Analysis of Real-World Pharmacovigilance Data

**DOI:** 10.3389/fcvm.2022.739044

**Published:** 2022-04-15

**Authors:** Javaria Ahmad, Aswani Thurlapati, Sahith Thotamgari, Udhayvir Singh Grewal, Aakash Rajendra Sheth, Dipti Gupta, Kavitha Beedupalli, Paari Dominic

**Affiliations:** ^1^Department of Medicine, Louisiana State University Health Sciences Center-Shreveport, Shreveport, LA, United States; ^2^Department of Medicine, Cardiology Service, Memorial Sloan Kettering Cancer Center, New York City, NY, United States; ^3^Department of Hematology and Oncology and Feist Weiller Cancer Center, Louisiana State University Health Sciences Center-Shreveport, Shreveport, LA, United States; ^4^Center of Excellence for Cardiovascular Diseases and Sciences, Louisiana State University Health Sciences Center-Shreveport, Shreveport, LA, United States

**Keywords:** chemotherapy, atrial fibrillation, cardiotoxicity, cardiac adverse events, FAERS

## Abstract

**Background:**

Several anti-cancer drugs have been linked to new onset atrial fibrillation (AF) but the true association of these drugs with AF is unknown. The FDA Adverse Event Reporting System (FAERS), a publicly available pharmacovigilance mechanism provided by the FDA, collects adverse event reports from the United States and other countries, thus providing real-world data.

**Objectives:**

To identify anti-cancer drugs associated with AF using the FAERS database.

**Methods:**

The FAERS database was searched for all drugs reporting AF as an adverse event (AE). The top 30 anti-cancer drugs reporting AF cases were shortlisted and analyzed. Proportional reporting ratio (PRR) was used to measure disproportionality in reporting of adverse events for these drugs.

**Results:**

When analyzed for AF as a percentage of all reported AE for a particular drug, Ibrutinib had the highest percentage (5.3%) followed distantly by venetoclax (1.6%), bortezomib (1.6%), carfilzomib (1.5%), and nilotinib (1.4%). The percentage of cardiac AE attributable to AF was also highest for ibrutinib (41.5%), followed by venetoclax (28.4%), pomalidomide (23.9%), bortezomib (18.2%), and lenalidomide (18.2%). Drugs with the highest PRR for AF included ibrutinib (5.96, 95% CI= 5.70–6.23), bortezomib (1.65, 95% CI = 1.52–1.79), venetoclax (1.65, 95% CI = 1.46–1.85), carfilzomib (1.53, 95% CI = 1.33–1.77), and nilotinib (1.46, 95% CI = 1.31–1.63).

**Conclusions:**

While newer anti-cancer drugs have improved the prognosis in cancer patients, it is important to identify any arrhythmias they may cause early on to prevent increased morbidity and mortality. Prospective studies are needed to better understand the true incidence of new onset AF associated with anti-cancer drugs.

## Introduction

The introduction of novel anti-cancer agents has significantly increased the survival in many malignancies, but none of these drugs is free of adverse effects. Rigorous identification of the toxicity profile of these drugs is crucial, given the better prognosis for patients treated with the newer agents. Cardiovascular toxicity is well established as a side effect of cancer therapy and is likely to be one of the most morbid of all adverse reactions. Although frequently reported as a part of the cardiovascular toxicity of anti-cancer drugs, cardiac arrhythmias, particularly atrial fibrillation (AF), have not been studied in a controlled manner (1).

AF is the most common cardiac arrhythmia, with an overall prevalence of 1–2% in the United States. The risk of AF increases with age, which is also a known risk factor for cancer (2). Management of AF in cancer patients can pose a unique challenge, particularly in hematological malignancies, where the risk of thrombogenesis has to be weighed against the risk of increased bleeding. Various anti-cancer agents have also been reported to cause new onset AF, but the true incidence is ambiguous. Although there is some emerging literature describing cancer treatment-induced arrhythmias, there is a dearth of real-world data exploring the association of AF with specific anti-cancer drugs (1). Ibrutinib is the only anti-cancer agent that has been relatively well studied with respect to the development of AF, with a reported incidence of 4–16% (3, 4) from clinical trials.

The importance of post-marketing surveillance of drugs cannot be emphasized enough as the real-world use of drugs and their adverse events (AE) differs from their use (and AE) in controlled clinical trials that are short-term and usually exclude vulnerable populations. FAERS is a publicly accessible international database containing AE reports submitted to the FDA by healthcare professionals, consumers, and manufacturers. We used the FAERS database to study anti-cancer drugs reported to have caused AF, to better understand the association of AF with these drugs.

## Methods

### Study Design and Data Source

A retrospective, observational, pharmacovigilance study was done on a de-identified publicly available FAERS dataset, which did not require IRB approval. FAERS complies with the international safety reporting guidance issued by the International Conference on Harmonization (ICH E2B). It collects AER submitted by healthcare professionals, consumers, and manufacturers around the world.

**Figure 1:**
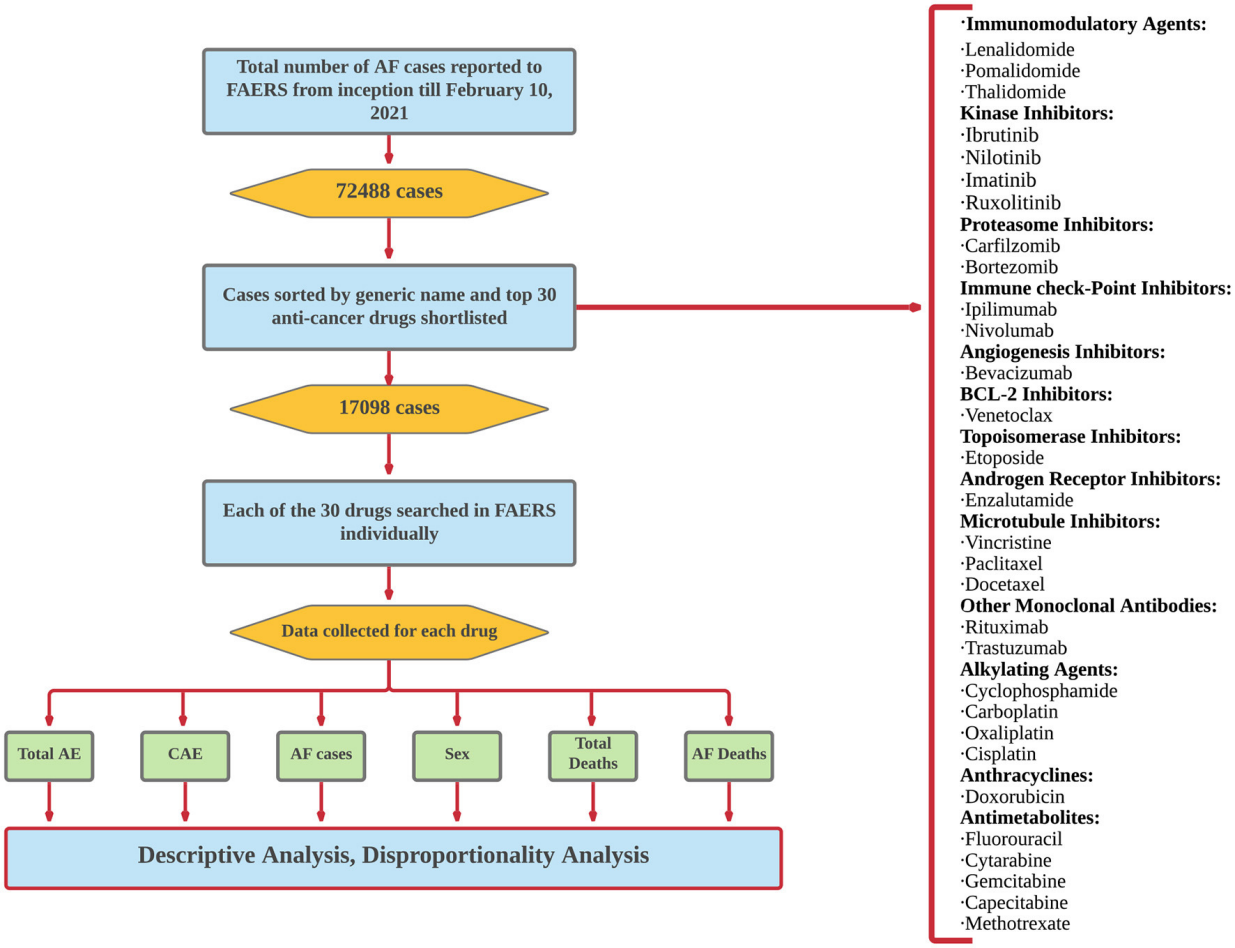
Algorithm of FAERS search and selection of anti-cancer drugs reported for AF.

The database was searched using the reaction term “Atrial Fibrillation.” All the reported cases of AF for each drug (queried by generic names) were collected from inception to February 10, 2021, and a shortlist created of the top 30 anti-cancer drugs based on reported AF cases. The selected drugs were then individually searched using the generic names and the reported AE were collected individually for each drug. Data were further narrowed down by applying various filters such as age, received year, sex, and outcomes (Figure 1).

### Statistical Methods and Analysis

First, a descriptive analysis of the data was performed. Then, a comparative analysis was done among the drugs in the group. The proportional reporting ratio (PRR) (5) was calculated for various parameters to determine the disproportionality in reporting. Data on patient characteristics and outcomes were collected and analyzed.

#### Descriptive Analysis

For each drug, AF cases were represented as a percentage of all AE as well as cardiac adverse events (CAE). In addition, AF cases by sex and by age were calculated as percentage of the total AF cases for the drug. The proportion of deaths among reported AF cases for each drug of interest was calculated. This proportion was then divided by the proportion of deaths among reported AF cases for all drugs that are reported to FAERS for AF to arrive at comparative mortality ratio (CMR).

#### Disproportionality Approach

PRR is a statistical tool used for quantitative signal detection in surveillance databases (6). PRR compares the frequency of reporting of an AE of a certain drug to the frequency of reporting of the same AE for other drugs in the reference group. A PRR >1 for a drug indicates that an AE is reported more frequently for the drug of interest relative to the drugs in the comparison group. Similarly, a PRR of 2 will suggest that the AE was reported twice as frequently for that drug compared to other drugs in the analysis and a PRR of 2 or more is generally considered significant disproportionality in AE reporting. We calculated the PRR of each drug for CAE, total AF cases, AF cases by sex, and total deaths in reported AF cases.

## Results

A total of 72,488 cases of AF were reported to FAERS. The top 30 anti-cancer drugs accounted for 17,098 of these cases (23.5% of total AF reports in FAERS). These drugs are listed in Table 1.

**Table 1:** The proportion of cases of AF, CAE, deaths, and PRR for all variables for all 30 drugs included in the analysis.

	**Drug**	**Total AEs reported**	**Total CAEs reported**	**Total AF cases**	**CAE as % of Total AE**	**PRR of CAE**	**AF as % of all AE of the drug**	**PRR of AF**	**AF as % of CAE of the drug**	**PRR of AF as % of CAE**	**PRR of AF in men**	**PRR of AF in women**	**Comparative mortality ratio**	**PRR of AF deaths**
1	Lenalidomide	231,623	12,954	2,363	5.59	0.695	1.02	1.027	18.24	1.479	1.115	1.127	1.154	1.012
2	Ibrutinib	40,151	5,149	2,138	12.82	1.688	5.32	5.961	41.52	3.532	6.953	4.126	0.887	4.625
3	Rituximab	109,507	9,130	1,225	8.33	1.086	1.12	1.131	13.42	1.042	0.82	1.255	1.27	1.222
4	Cyclophosphamide	104,111	9,110	959	8.75	1.143	0.92	0.919	10.53	0.804	0.722	1.07	1.796	1.015
5	Paclitaxel	71,106	6,934	762	9.75	1.278	1.07	1.078	10.99	0.844	0.823	1.594	1.636	1.409
6	Doxorubicin	73,947	8,183	749	11.06	1.462	1.01	1.017	9.15	0.695	0.752	1.289	1.765	1.216
7	Bortezomib	37,496	3,337	609	8.89	1.157	1.62	1.652	18.25	1.428	1.653	1.326	1.526	1.728
8	Carboplatin	58,634	4,999	599	8.52	1.108	1.02	1.026	11.98	0.925	0.989	1.166	1.702	1.347
9	Pomalidomide	49,099	2,421	579	4.93	0.632	1.18	1.189	23.92	1.882	1.362	1.274	1.005	0.941
10	Methotrexate	12,9001	7,000	578	5.42	0.686	0.45	0.43	8.26	0.627	0.255	0.738	1.372	0.886
11	Cisplatin	52,205	4,522	574	8.66	1.126	1.1	1.106	12.69	0.982	1.463	0.707	1.961	1.445
12	Bevacizumab	72,696	4,998	558	6.87	0.886	0.77	0.762	11.16	0.86	0.687	0.864	1.218	0.496
13	Fluorouracil	57,638	5,459	521	9.47	1.237	0.9	0.904	9.54	0.731	0.989	0.872	1.174	0.948
14	Docetaxel	68,681	4,045	410	5.88	0.755	0.6	0.589	10.14	0.78	0.625	0.651	1.732	1.219
15	Oxaliplatin	42,595	3,960	388	9.29	1.21	0.91	0.912	9.8	0.753	1.062	0.749	1.616	1.348
16	Cytarabine	38,000	3,609	376	9.49	1.237	0.99	0.992	10.42	0.802	1.098	0.67	2.712	1.442
17	Nivolumab	47,764	3,174	362	6.64	0.857	0.76	0.755	11.41	0.881	1.023	0.535	1.836	0.639
18	Thalidomide	37,592	3,163	353	8.41	1.092	0.94	0.941	11.16	0.861	0.983	0.961	1.798	0.538
19	Etoposide	41,950	3,569	342	8.50	1.105	0.82	0.814	9.58	0.737	0.983	0.502	2.032	0.891
20	Nilotinib	22,347	3,496	325	15.64	2.054	1.45	1.468	9.3	0.714	1.705	1.371	0.906	1.028
21	Capecitabine	59,896	3,890	291	6.49	0.836	0.49	0.478	7.48	0.572	0.488	0.505	2.284	0.522
22	Venetoclax	16,683	958	273	5.74	0.742	1.64	1.652	28.5	2.226	2.171	1.216	1.024	0.709
23	Imatinib	49,904	3,241	260	6.49	0.837	0.52	0.515	8.02	0.615	0.535	0.582	1.395	0.259
24	Ruxolitinib	38,969	1,917	241	4.91	0.632	0.62	0.615	12.57	0.973	0.255	0.279	1.661	0.797
25	Trastuzumab	33,628	4,904	240	14.58	1.923	0.71	0.712	4.89	0.37	0.057	1.666	1.668	1.135
26	Vincristine	30,753	2,129	225	6.92	0.895	0.73	0.73	10.57	0.816	0.584	0.725	1.679	0.769
27	Enzalutamide	44,071	1,776	214	4.02	0.515	0.49	0.481	12.05	0.932	0.912	0	1.377	0.421
28	Ipilimumab	23,455	1,313	206	5.59	0.722	0.88	0.879	15.69	1.217	1.274	0.537	1.76	1.029
29	Carfilzomib	12,437	1,565	190	12.58	1.637	1.53	1.538	12.14	0.939	1.81	0.802	1.551	2.066
30	Gemcitabine	18,915	1,488	188	7.86	1.019	0.99	0.997	12.63	0.978	1.029	0.883	2.009	1.315

### Anti-cancer Drugs and Cardiac Adverse Events (CAE)

**Figure 2:**
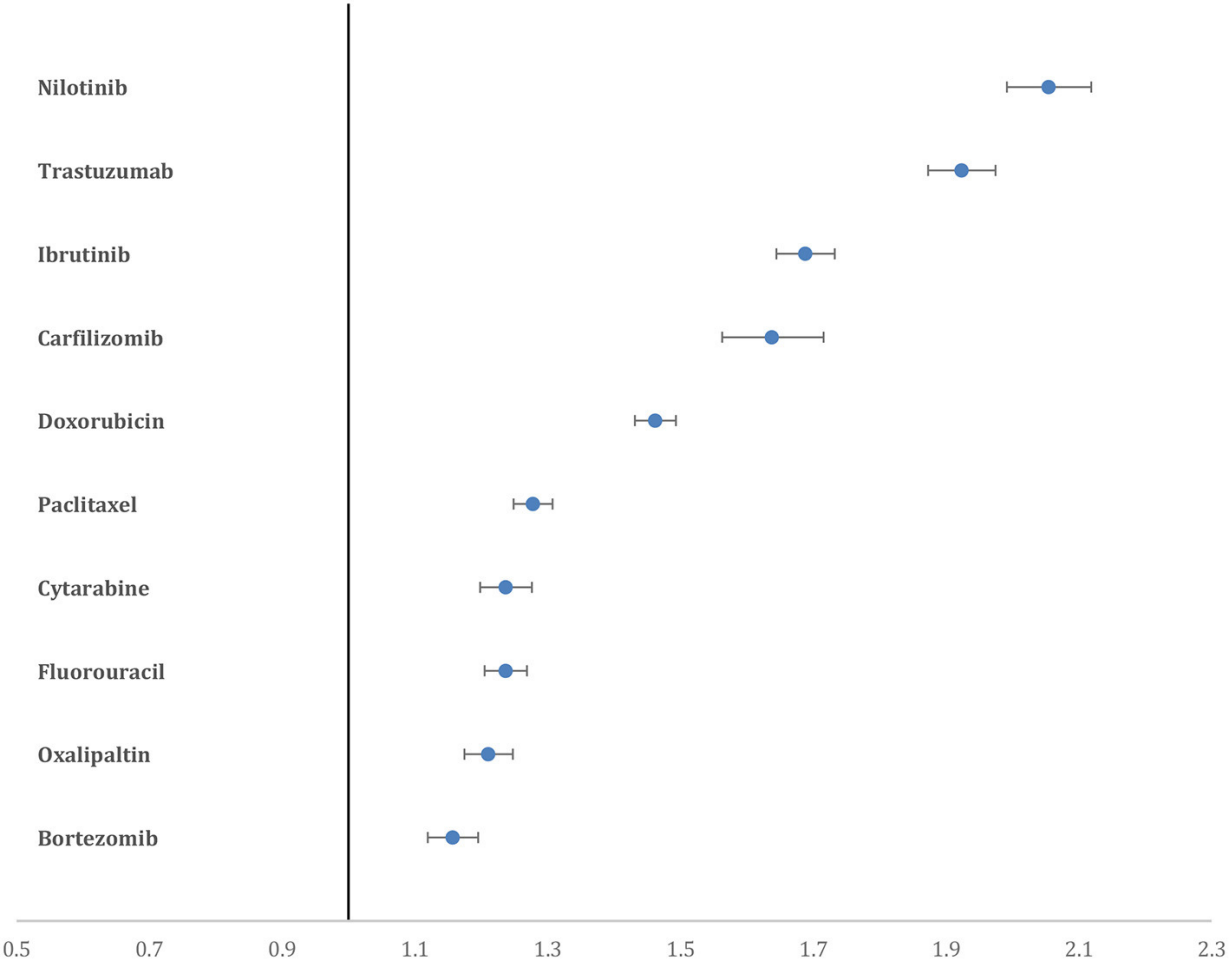
Forest plot showing the PRR of CAE for the top 10 drugs. Nilotinib and trastuzumab have higher PRR compared to all other drugs.

Nilotinib had the highest proportion of CAE, which accounted for 15.6% of the total reported AE of the drug, followed by trastuzumab (14.5%), ibrutinib (12.8%), carfilzomib (12.5%), and doxorubicin (11%). Nilotinib had the highest PRR for CAE (2.05, 95% CI = 1.99–2.11), followed by trastuzumab (1.92, 95% CI = 1.87–1.97), ibrutinib (1.68, 95% CI = 1.64–1.73) and carfilzomib (1.63, 95% CI = 1.56–1.71) (Figure 2).

### Anti-cancer Drugs and AF

**Figure 3:**
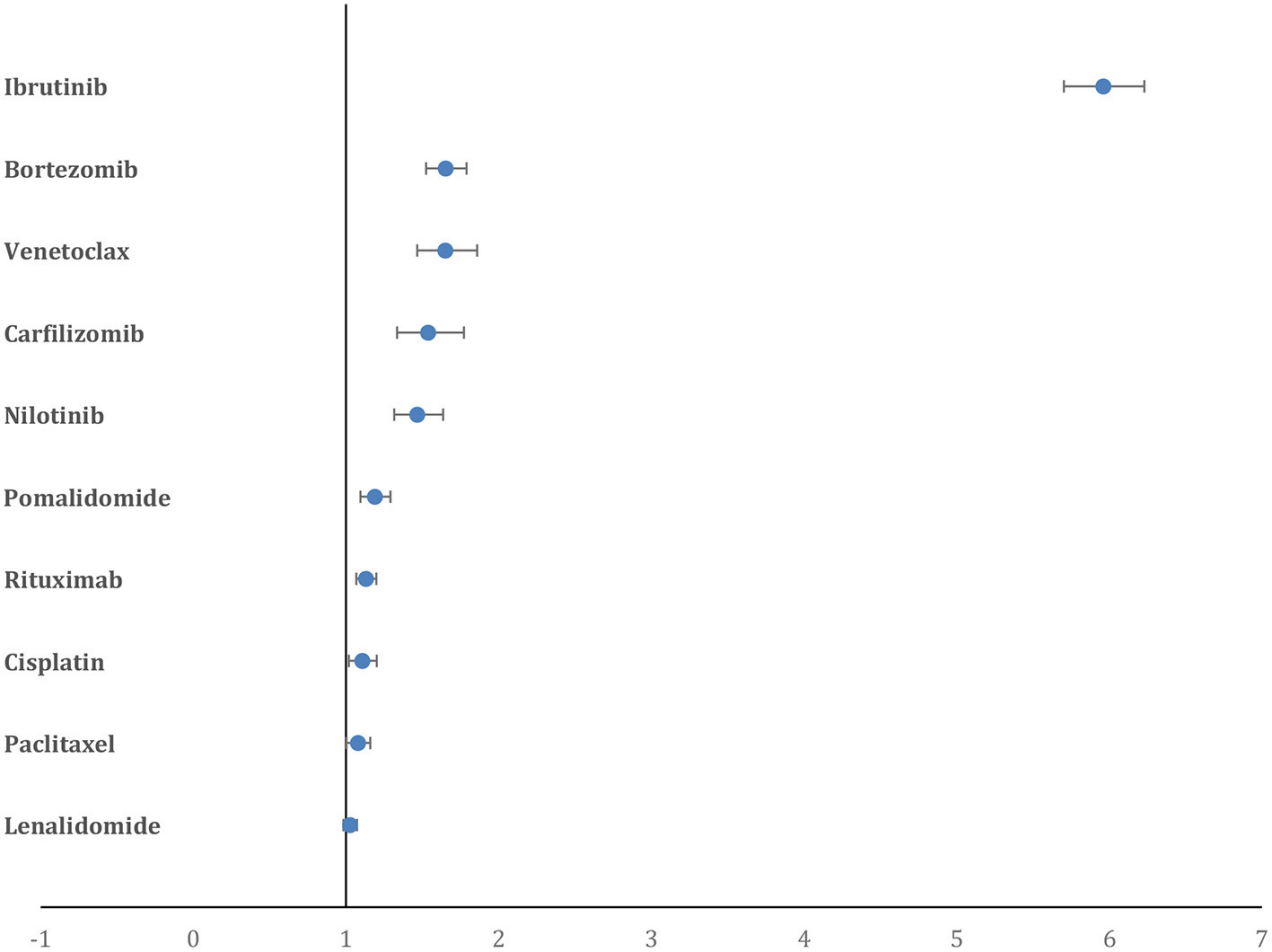
Forest plot showing the PRR of AF for the top 10 drugs. Ibrutinib has a significantly higher PRR compared to all other drugs.

When analyzed for AF as a percentage of all reported AE for a particular drug, Ibrutinib had the highest percentage (5.3%) followed distantly by venetoclax (1.6%), bortezomib (1.6%), carfilzomib (1.5%), and nilotinib (1.4%). Similarly, the percentage of CAE attributable to AF was also highest for ibrutinib (41.5%), followed by venetoclax (28.4%), pomalidomide (23.9%), bortezomib (18.2%), and lenalidomide (18.2%). Drugs with the highest PRR for AF included ibrutinib (5.96, 95% CI = 5.70–6.23), bortezomib (1.65, 95% CI = 1.52–1.79), venetoclax (1.65, 95% CI = 1.46–1.86), carfilzomib (1.53, 95% CI = 1.33–1.77), and nilotinib (1.46, 95% CI = 1.31–1.63) (Figure 3).

The PRR for AF in men was highest for ibrutinib (6.95, 95% CI = 6.55–7.37), followed by venetoclax (2.17, 95% CI = 1.87–2.51), carfilzomib (1.81, 95% CI = 1.50–2.17), nilotinib (1.70, 95% CI = 1.47–1.96), and bortezomib (1.65, 95% CI = 1.47–1.84) (Supplementary Figure 1). Similarly, PRR for AF in women was also highest for ibrutinib (4.12, 95% CI = 3.78–4.49), followed by trastuzumab (1.66, 95% CI = 1.45–1.91), paclitaxel (1.59, 95% CI = 1.44–1.76), nilotinib (1.37, 95% CI = 1.13 −1.65), and bortezomib (1.32, 95% CI = 1.14–1.53) (Supplementary Figure 2).

### Deaths in Patients Reported to Have AF During Anti-cancer Therapy

**Figure 4:**
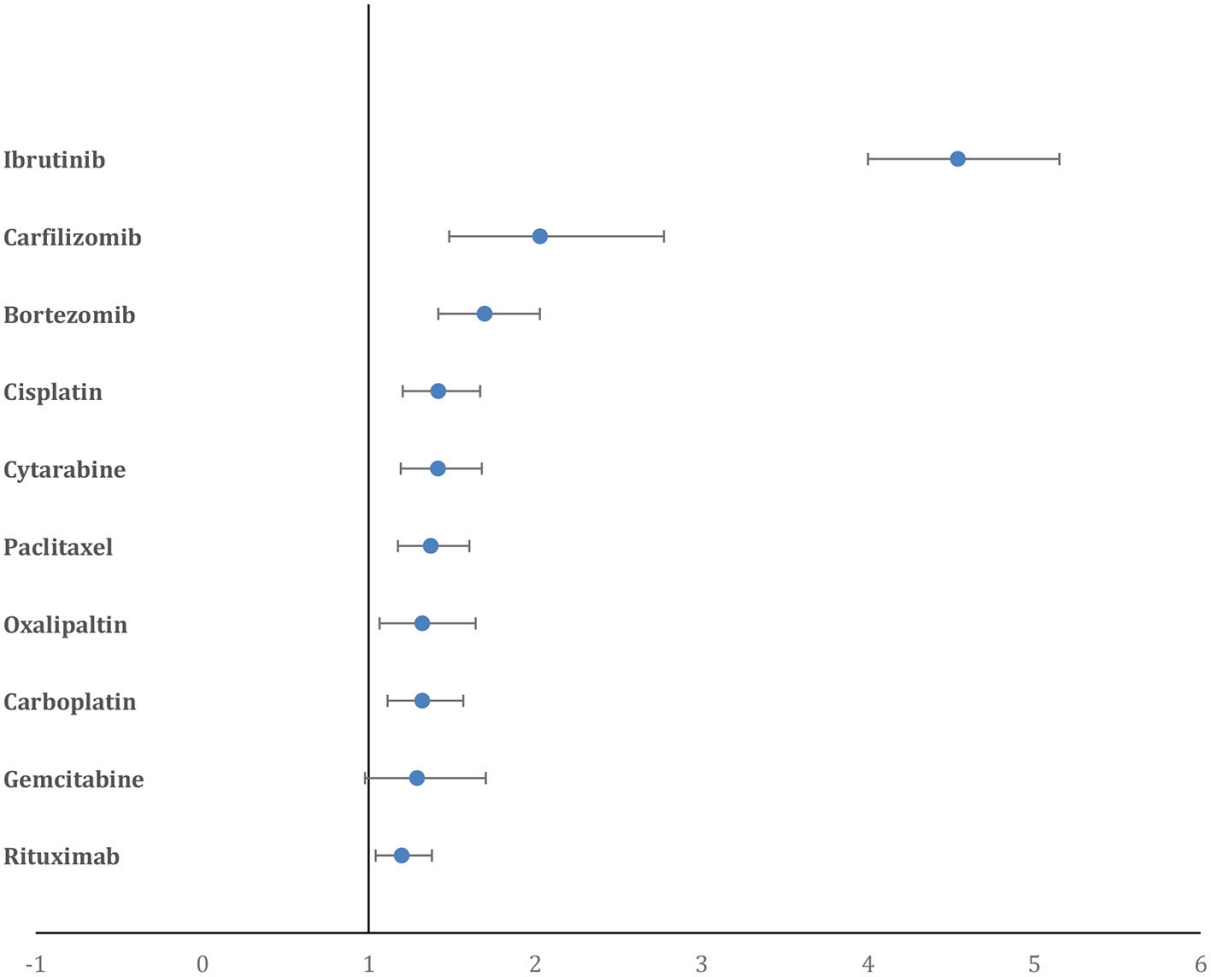
Forest plot showing the PRR of deaths in AF cases for the top 10 drugs. Ibrutinib has a significantly higher PRR compared to all other drugs.

The percentage of reported AF cases in patients who died, of the total number of deaths reported for a drug, was highest for cytarabine (35.9%), followed by capecitabine (30.2%), etoposide (26.9%), gemcitabine (26.5%) and cisplatin (25.9%). Paralleling this, the comparative mortality ratio (CMR) was highest for cytarabine (2.7), followed by capecitabine (2.2), etoposide (2.03), gemcitabine (2) and cisplatin (1.9). However, when assessed using disproportional reporting measures, the PRR for deaths in reported AF cases was highest for ibrutinib (4.62, 95% CI = 4.07–5.24), followed by carfilzomib (2.06, 95% CI = 1.51–2.82), bortezomib (1.72, 95% CI = 1.44–2.06), cisplatin (1.44, 95% CI = 1.22–1.70), and cytarabine (1.44, 95% CI = 1.21–1.71) (Figure 4).

## Discussion

### Key Results

**Figure 5:**
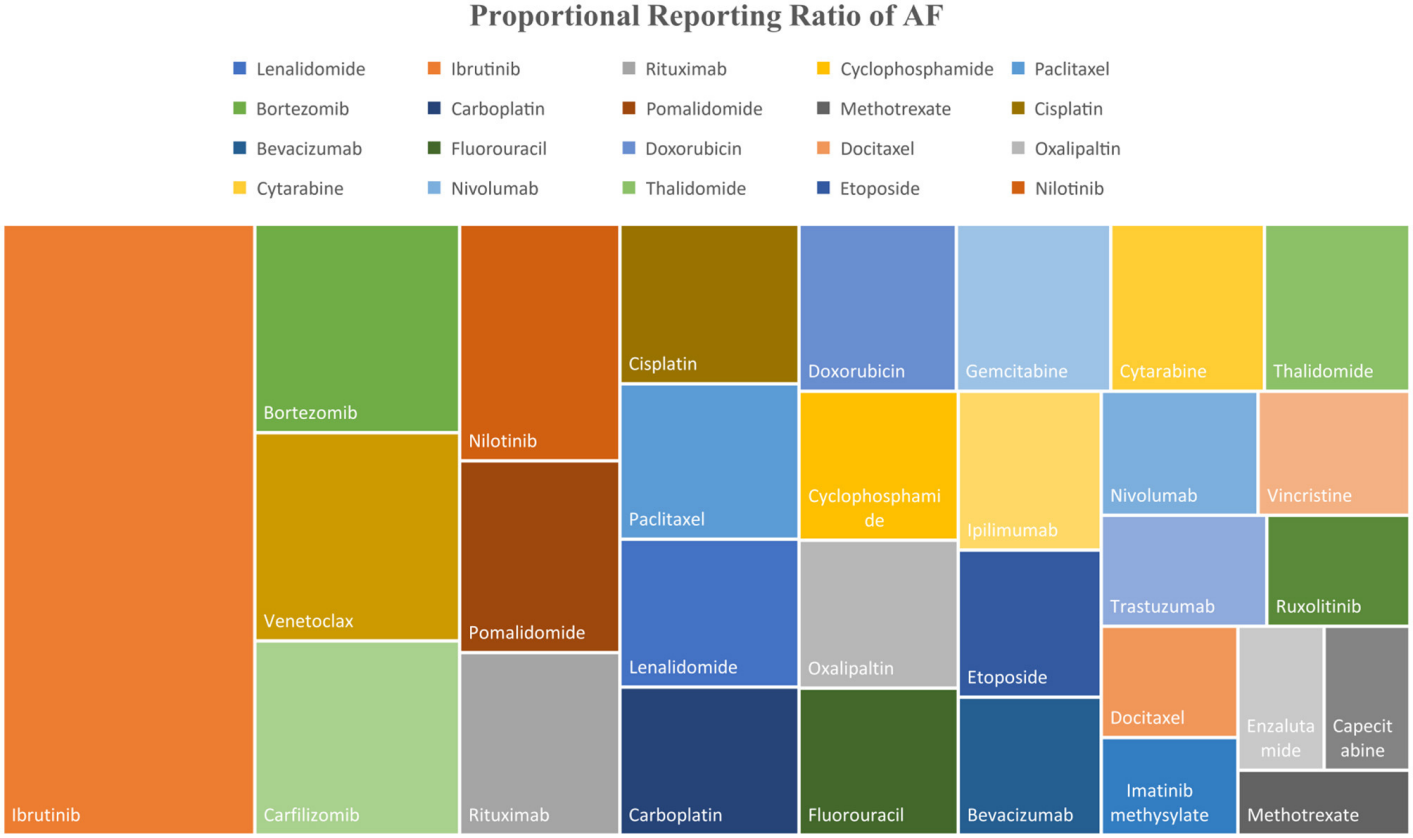
Tree map showing the PRR of AF for top 30 anti-cancer drugs included in the analysis.

Our comparison of anti-cancer drugs using the PRR to assess the signal-to-noise ratio of public reporting to FAERS of cardiac-related AE showed that nilotinib, ibrutinib, trastuzumab, and carfilzomib had the most CAE reported, while ibrutinib, venetoclax, bortezomib, carfilzomib, and nilotinib had the highest AF cases reported when compared to other drugs in the analysis (Figure 5). The drugs with highest reported deaths among AF cases were ibrutinib, bortezomib, carfilzomib, cisplatin, and cytarabine.

Atrial fibrillation in cancer patients has been shown to be associated with poor outcomes (7). The incidence of thromboembolism in cancer patients with new onset AF increases two-fold (8). In addition, various anti-cancer drugs increase the risk of thrombogenesis (9). Finally, the risk of bleeding in cancer patients on anticoagulants is higher than that in patients who do not have cancer (10). All these factors make the management of AF in cancer patients a unique challenge, underscoring the relevance of our findings identifying newer anti-cancer drugs that are disproportionately reported to FAERS for AF as an AE.

### Anti-cancer Therapy and AF

A variety of modalities of cancer treatment, including chemotherapy, immunotherapy, hormone and radiation therapy, and surgical resection, have each been separately associated with AF. Various anti-cancer drugs, including ibrutinib, doxorubicin, and cisplatin, amongst others, have been associated with AF (11). We have previously established that radiation therapy may be an independent risk factor for AF development (12). Postoperative AF has been associated with surgical resection of lung, colorectal, and esophageal tumors (13–15). Patients with cancer often receive multiple treatment modalities and multiple chemotherapeutic agents. The amalgamation of conventional risk factors, cancer itself, and cancer treatment creates a unique scenario in which the assessment of the specific AF risk with a certain drug can be challenging. Below, we briefly discuss some of the drugs from our analysis that had significantly disproportional reporting for AF and what is known in terms of their association with AF.

### Ibrutinib and Nilotinib

Ibrutinib is an irreversible Bruton's tyrosine kinase inhibitor that prevents downstream activation of B-cells. It is currently used in the treatment of chronic lymphocytic leukemia (CLL), mantle cell lymphoma, marginal zone lymphoma, small lymphocytic lymphoma, and the plasma cell disorder Waldenstrom's macroglobulinemia. An AF incidence of 6% was noted for ibrutinib in the landmark RESONATE and RESONATE-2 trials that led to the FDA approval of the drug (16). A meta-analysis by Yun et al. (17) of AF in patients treated with ibrutinib vs. other chemo-immunotherapeutic agents for B-cell malignancies showed an approximately nine-fold increased risk of AF following treatment with ibrutinib. Our results showing the highest AF PRR for ibrutinib (5.96, 95% CI = 5.70–6.23) are in line with the known evidence for AF and the drug. Based on a risk prediction model, the risk of AF in patients treated with ibrutinib may be modified by co-existent conditions, including age >65 years, male sex, valvular heart disease, cardiomyopathy, thyroid abnormality, chronic lung disease, diabetes mellitus, and grade-3 infections (18). The precise mechanism behind the pathogenesis of AF in connection with ibrutinib treatment is unknown.

Nilotinib is another member of the TKI family used in the treatment of CML (19), that is known to be highly vasculotoxic and cardiotoxic. No prior evidence of an association between AF and nilotinib exists in the literature. In a Phase II trial in which 73 patients treated with nilotinib were followed for 6 years, only 1 developed AF. Similarly, another study reported that AF occurred in 1 of 81 patients treated with nilotinib (20). Another study of the cardiotoxicity of TKI in the FAERS database reported a significantly increased adjusted Reporting Odds Ratio (aROR) for cardiac arrhythmias following treatment with nilotinib (2.7), but did not specify the type of arrhythmia (21). While our study highlights a potential novel association, further prospective studies are warranted to confirm the association and determine the causation.

### Bortezomib and Carfilzomib

Bortezomib and carfilzomib are both proteasome inhibitors (PI) that act by inhibiting the NF-κB pathway on multiple myeloma (MM) cells leading to cell death. The available data on the incidence of AF in connection with these drugs are scarce and inconsistent. A study of four Phase II clinical trials of carfilzomib showed that cardiac arrhythmias developed in 13.3% of the patients (*n* = 526); however, 73.6% of the patients enrolled had a prior history of cardiovascular events (22). Another meta-analysis of cardiovascular adverse events related to treatment with carfilzomib reported cardiac arrhythmias in 2.4% of patients from a total of 13 studies (23). A smaller prospective study (*n* = 95) compared the CAE that resulted from treatment with carfilzomib to those of bortezomib. Results showed that the CAE were significantly higher in patients treated with carfilzomib than in those treated with bortezomib (51% vs. 17% respectively), consistent with our results (PRR for CAE: carfilzomib, 1.63 (95% CI = 1.56–1.71) vs. bortezomib, 1.15 (95% CI = 1.11–1.19). The incidence of AF was comparable for the two drugs, with 2 of 65 patients treated with carfilzomib and 1 of 30 patients treated with bortezomib developing AF (24). Similar to these results, we found a comparable increase in the reporting of AF for both bortezomib and carfilzomib [1.65 (95% CI = 1.52–1.79) and 1.53 (95% CI = 1.33–1.77), respectively].

One difficulty in differentiating between these two drugs and their association with AF is that carfilzomib is mostly reserved for relapsed or refractory MM and most of the patients receiving carfilzomib had already been treated with bortezomib (25). Bortezomib is one of the newer anti-cancer drugs identified for its association with AF by Alexandre et al. in their pharmacovigilance study based on the World Health Organization's Vigibase AE dataset; however, any association of AF with carfilzomib was not reported (26). Even though our study highlights a potential association between proteasome inhibitors and AF, the results should be looked upon with caution. Due to lack of comorbidity data and temporal association, causation and definitive conclusions cannot be drawn. An important confounding factor in these drugs is the high prevalence of cardiac disease in MM patients due to increased age, hyper-viscosity, arteriovenous shunts, anemia, and amyloidosis (27). It cannot be determined whether the AF was caused by the drugs alone, or by the combination of all other factors, but the over-reporting in studies from two different databases merits further investigation.

### Venetoclax and Pomalidomide

Venetoclax is a B-cell lymphoma (BCL-2) homology domain-3 (BH-3) mimetic, selective B-cell lymphoma (BCL-2) inhibitor used in the treatment of lymphomas and leukemias (28). We found increased reporting of AF with venetoclax (PRR 1.65, 95% CI = 1.46–1.85). It is difficult to positively identify the association of venetoclax with AF, because it is used either as first line for the treatment of CLL or used subsequently after progression on ibrutinib, that is known for its association with AF. A Phase II RCT investigating the combination of ibrutinib and venetoclax used to treat high-risk elderly CLL patients showed an incidence of AF in 15% of the patients (29) that is nearly the same as that reported for ibrutinib (30), providing strong evidence for ibrutinib as the cause of AF rather than venetoclax (29). No studies have reported on the incidence of AF during treatment with venetoclax alone.

Pomalidomide is a novel immunomodulatory agent used in the treatment of MM. Prior to our study, pharmacovigilance analysis of the Vigibase dataset showed that AF was reported to occur following treatment with pomalidomide (26). It should be noted that this drug is approved for use in treating MM that is refractory to at least two prior therapies, including lenalidomide and bortezomib, both of which have been reported to be associated with AF. It is important to note that patients with MM are at increased risk of AF in the first place due to age and high cardiovascular disease burden. While there is increased reporting of AF with the above-mentioned drugs, the results should be interpreted with caution as the association does not imply causation.

### Mortality Associated With Reported AF Cases

AF increases the risk of heart failure, bleeding complications, myocardial infarction, and all-cause mortality (7, 31). Although a large number of AF patients reported to be on treatment with cytarabine (35.9%), etoposide (26.9%), and gemcitabine (26.5%) died compared to AF patients being treated with other chemotherapeutic agents, this could reflect the severity of the cancer being treated with these drugs rather than an association with the AF reported for the drug. These traditional chemotherapeutic drugs are commonly used to treat cancers with unfavorable survival rates such as AML, pancreatic cancer, bladder cancer, metastatic lung cancer, and ovarian cancer. Therefore, to refine the signal-to-noise ratio, we calculated the PRR for deaths in patients with reported AF. Ibrutinib had a significantly higher PRR for deaths in patients with reported AF, almost three-fold higher than the PRR for the next highest anti-cancer drug, carfilzomib. This suggests that the adverse events related to AF with Ibrutinib could be associated with increased mortality and merits further prospective studies.

### Evidence Concerning AF and Anti-cancer Drugs: Vigibase vs. FAERS

Recently, a pharmacovigilance study by Alexandre et al. (26) based on the Vigibase dataset identified 19 anti-cancer drugs associated with AF. Of the 19 drugs identified, new associations with AF were reported for nine drugs including lenalidomide, pomalidomide, nilotinib, ponatinib, midostaurin, azacytidine, clofarabine, docetaxel, and obinutuzumab. Our results confirm the over-reporting of AF with lenalidomide, pomalidomide, and nilotinib, but our PRR for docetaxel was <1. In addition, ponatinib, midostaurin, azacytidine, clofarabine, and obinutuzumab were not included in our analysis due to the small number of cases reported to FAERS. For example, midostaurin has had no AF cases reported to FAERS, and the study by Alexandre et al. had only 20 AF cases. On the other hand, we found significant over-reporting of cases for AF following use of carfilzomib and venetoclax.

In general, FAERS had a higher number of total AF cases reported for most of the chemotherapeutic drugs compared to reports in Vigibase, which is a global database. In addition, we used a different quantitative signal detection tool than Alexandre et al. used; in their study, the Reporting Odds Ratio (ROR) was used as a measure of disproportionality analysis, but on the other hand we used PRR. While PRR and ROR are two different measures and have their own advantages and drawbacks, both are authentic qualitative signal detection tools (5). Another difference is that Alexandre et al. adjusted the ROR for age, sex, geographical region, and comorbidities, whereas inconsistent reporting in FAERS prevented us from performing such adjustments.

## Limitations

Our study had limitations inherent to pharmacovigilance design. First, since there was no reported temporal relationship between the incidence of AF or the outcomes and the specified drug, causation could not be established. To overcome this, we calculated the PRR to detect the disproportionality in reporting and to aid in quantitative signal detection. Second, it is possible that both under-reporting and over-reporting occurred due to missing and duplicate reports. Reported adverse event data cannot be used to calculate the incidence of AF in the population; instead, it should be used to identify the potential hazards of the drug. Third, we did not have data on co-morbidities of the patients to calculate the adjusted reporting ratio. Finally, we shortlisted the drugs by the number of AF cases reported to FAERS. Therefore, comparatively newer drugs, or infrequently used drugs, which might have been associated with AF but had inadequate cases reported to FAERS, could have been missed in our analysis.

## Conclusion

Atrial fibrillation associated with cancer therapy has lately attracted the attention of the cardio-oncology community, but there is no real data available to help guide the detection, monitoring, and treatment of AF in these patients. Our analysis of the reported cases to FAERS has uncovered anti-cancer drugs that were previously less known for their association with AF. Focused prospective studies should be conducted to gain a better understanding of the true incidence of AF associated with these anti-cancer agents.

## Data Availability Statement

Publicly available datasets were analyzed in this study. This data can be found here: https://www.fda.gov/drugs/questions-and-answers-fdas-adverse-event-reporting-system-faers/fda-adverse-event-reporting-system-faers-public-dashboard.

## Author Contributions

PD and JA: study conception and design. JA and AT: data collection. ST: statistical analysis. JA with input from all authors: draft preparation. UG, AS, KB, DG, and PD: critical revision. All authors: interpretation of results. All authors reviewed and approved the final version of manuscript.

## Funding

This publication was supported by an Institutional Development Award from the National Institutes of General Medical Sciences of the National Institutes of Health (NIH) under Grant Number P20GM121307 to C.G. Kevil.

## Conflict of Interest

The authors declare that the research was conducted in the absence of any commercial or financial relationships that could be construed as a potential conflict of interest.

## Publisher's Note

All claims expressed in this article are solely those of the authors and do not necessarily represent those of their affiliated organizations, or those of the publisher, the editors and the reviewers. Any product that may be evaluated in this article, or claim that may be made by its manufacturer, is not guaranteed or endorsed by the publisher.
