# Supplementary material for: Anti-cancer Drugs Associated Atrial Fibrillation—An Analysis of Real-World Pharmacovigilance Data
Source: Front Cardiovasc Med. 2022 Apr 15;9:739044. doi: 10.3389/fcvm.2022.739044 (PMC9051026; doi:10.3389/fcvm.2022.739044)
Supplement: Supplementary file 1 [file Data_Sheet_1.docx]

**Supplementary Figure Legends**

**Figure 1.** Forest plot showing the PRR of AF in men for the top 10 drugs. Ibrutinib has a significantly higher PRR compared to all other drugs.

**Figure 2.** Forest plot showing the PRR of AF in women for the top 10 drugs. Ibrutinib has a significantly higher PRR compared to all other drugs.

**Supplementary figures**

**Figure 1. PRR for AF in Men**

**Figure 2. PRR for AF in women**
